# Fermented Goat Milk Consumption Enhances Brain Molecular Functions during Iron Deficiency Anemia Recovery

**DOI:** 10.3390/nu11102394

**Published:** 2019-10-07

**Authors:** Jorge Moreno-Fernández, Inmaculada López-Aliaga, María García-Burgos, María J.M. Alférez, Javier Díaz-Castro

**Affiliations:** 1 Department of Physiology, Faculty of Pharmacy, Campus Universitario de Cartuja, E-18071 Granada, Spain; jorgemf@ugr.es (J.M.-F.); milopez@ugr.es (I.L.-A.); mariagb@ugr.es (M.G.-B.); javierdc@ugr.es (J.D.-C.); 2 Institute of Nutrition and Food Technology “José Mataix Verdú”, University of Granada, E-18071 Granada, Spain

**Keywords:** iron deficiency anemia, fermented goat milk, brain molecular functions, neuroprotective effect

## Abstract

Iron deficiency anemia (IDA) is one of the most prevalent nutritional deficiencies worldwide. Iron plays critical roles in nervous system development and cognition. Despite the known detrimental consequences of IDA on cognition, available studies do not provide molecular mechanisms elucidating the role of iron in brain functions during iron deficiency and recovery with dairy components. In this study, 100 male Wistar rats were placed on a pre-experimental period of 40 days and randomly divided in two groups: a control group receiving a normal-Fe diet, (45 mg/kg), and an Fe-deficient group receiving a low-Fe diet (5 mg/kg). At day 40, 10 rats per group were sacrificed to anemia control, and 80 rats were divided into eight experimental groups fed with fermented goat or cow milk-based diets, with normal Fe content or Fe overload (450 mg/kg) for 30 days. IDA decreased most of the parameters related to brain molecular functions, namely dopamine, irisin, MAO-A, oxytocin, β-endorphin, and α-MSH, while it increased synaptophysin. These alterations result in an impairment of brain molecular functions. In general, during anemia recovery, fermented goat milk diet consumption increased dopamine, oxytocin, serotonin, synaptophysin, and α-MSH, and decreased MAO-A and MAO-B, suggesting a potential neuroprotective effect in brain functions, which could enhance brain molecular functions.

## 1. Introduction

Despite the global progress achieved in nutrition and science development, iron deficiency (ID) remains the most prevalent nutritional deficiency worldwide, affecting between 4–6 billion people. ID is also the main cause of anemia. Two billion people, more than 25% of the world’s population, has manifestations of iron deficiency anemia (IDA) [1]. Among its many physiological functions, iron plays key roles in nervous system development and function, via biochemical processes involved in brain structure and functions. There are several studies on the effects of ID on brain development and functions [2,3], and on the link between iron status and cognitive function [4,5]. In this sense, iron is involved in the adequate myelination of the white matter, hippocampus development, and neurotransmitter homeostasis, with neurophysiological and behavioral outcomes showing relations to iron status at several life stages [6]. Behavioral performance and brain functions, as measured by electroencephalography, are sensitive to iron status [5].

Moreover, several studies showed that anemia may be associated with impairment in cerebral blood flow and oxygen metabolism, impaired cognitive function, confusion, loss of concentration, impaired memory, and low mental alertness [7]. Acute anemia results in the slowing of data-processing ability and memory impairment in humans [8]. In addition, cognitive ability is improved by erythropoietin [9].

Recent studies found several evidences that iron supplementation improved attention and concentration [5,10], as well as cognitive function [4]. These improvements in psychological symptoms appear to be mediated by an enhancement of oxygen transport to the brain [11].

In the scientific literature, raised awareness about the effects of nutrition on brain molecular functions exists, although few studies have examined the cognitive effects of fermented milks on cognitive function, and none of them linked to iron status. Currently, dairy products are increasingly consumed to prevent cognitive impairment and dementia [12]. Recent evidence suggests that the inclusion of dairy products in a balanced diet might have several positive effects on cognitive health in advanced age [13].

On the other hand, we have reported previously that fermented goat milk consumption is beneficial in IDA due to the improvement in the key proteins of intestinal iron metabolism, enhancing the digestive and metabolic utilization of iron, increasing iron deposits in target organs, and favoring the recovery of hematological parameters [14]. However, despite the known detrimental consequences of IDA on cognition, research linking iron status to brain functions has largely been ignored, and available studies are mainly focused on psychological tests evaluating cognitive function, concentration, memory, and alertness without providing molecular mechanisms elucidating the role of iron in brain functions during ID and recovery with dairy components. Hence, taking into account all these considerations, we set out to investigate the impact of fermented goat or cow milk-based diets (with normal or overload iron content) consumption during IDA recovery on the brain molecular functions in animal models.

## 2. Materials and Methods

### 2.1. Fermentation and Dehydration of the Milks

Fermented milks were prepared following the previously described method [15].

### 2.2. Animals 

One hundred male *Wistar* albino breed rats (3 weeks of age and weighing about 34.56 ± 6.35 g) were included in this study. Experimental procedures were carried out in agreement with the guidelines about animal welfare and experimentation (Declaration of Helsinki; Directive 2010/63/EU). 

Individual, ventilated, and thermos-regulated cages were used to randomly allocate the rats. 

The room temperature (22.5 ℃), humidity (60%), and a 12-h light-dark cycle were automatically controlled. Animal weight was taken as a variable factor, and numbers randomly assigned were generated (Microsoft Excel, 2016 (Microsoft, Redmond, WA, USA). Subsequently, the mean of the 100 weights were compared by a one-way ANOVA. The groups were formed to obtain a probability level of *p* = 0.09. The animals of each group had initial weights of 34.28 ± 5.12 g, 34.39 ± 5.01 g, 36.27 ± 4.58 g, 35.23 ± 6.12 g, 36.13 ± 6.37 g, 35.29 ± 6.01 g, 36.13 ± 5.13 g, 37.92 ± 6.21 g, 33.88 ± 6.11 g and 35.53 ± 5.76 g (*p* > 0.05), respectively. Diet intake was controlled, pair feeding all the animals, and bidistilled water was available ad libitum. 

### 2.3. Experimental Design 

**Figure 1:**
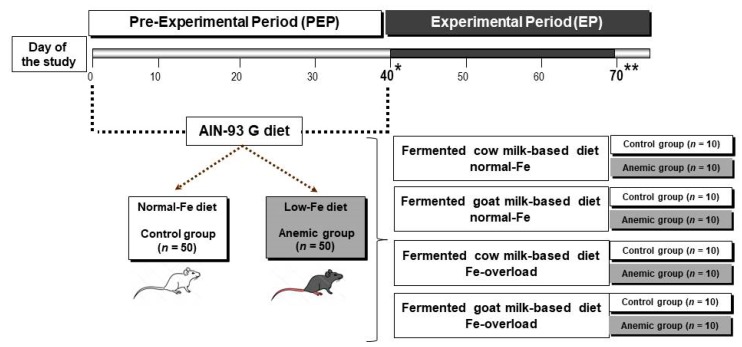
The study experimental design. * 10 control and 10 anemic rats were totally bled out by cannulation of the abdominal aorta. ** 80 animals were totally bled out by cannulation of the abdominal aorta. AIN-93 G diet, Standard diet from the American Institute of Nutrition (for the growth phase).

Figure 1 features the experimental design of the study. During the pre-experimental period (PEP), (*n* = 100) rats were divided into two groups: the control group receiving the AIN 93G diet with a normal Fe diet (*n* = 50, 44.8 mg/kg by analysis) [16], and the anemic group receiving the same diet, but with a low Fe content (*n* = 50, 6.1 mg/kg by analysis), induced experimentally during 40 days by a method developed previously by our research group [17]. On day 40 of the study, 2 aliquots of blood were collected from the caudal vein of each rat. One of them had Ethylenediaminetetraacetic acid (EDTA) to measure all the hematological parameters, and the other one was centrifuged (1500× *g*, 4 ℃, 15 min) without anticoagulant to separate the serum and subsequent analysis of serum iron, total iron-binding capacity (TIBC), ferritin, and hepcidin.

When the induction of the anemia period finalized (day 40 of the study), 10 rats per group were sacrificed, and the remaining 80 animals subsequently started the experimental period (EP) in which the control (*n* = 40) and anemic groups (*n* = 40) were further fed for 30 days with a fermented cow milk or fermented goat milk-based diet, with normal Fe content (45 mg·kg^−1^) or Fe overloaded content (450 mg·kg^−1^) to induce chronic Fe overload [18] prepared with fermented cow or goat milk, as previously reported [14].

At the end of the PEP and the end of EP, 20 and 80 animals respectively were anesthetized intraperitoneally with sodium pentobarbital (Sigma-Aldrich Co., St. Louis, MO, USA) and totally bled out. Blood aliquots with EDTA were analyzed to measure the hematological parameters, and the rest of the blood was centrifuged (1500× *g*, 4 ℃, 15 min) without anticoagulant to separate the red blood cells from the serum and to determine the parameters related to iron status. The brain was removed immediately, weighed, and was split into two portions (which included parts of both hemispheres) and placed on cold saline buffer. Brain samples were homogenized in phosphate-buffered saline (PBS), pH 7.4 by the homogenizer. Protease inhibitor cocktail (Sigma-Aldrich Co., St. Louis, MO, USA) was used. The homogenate was centrifuged at 2000× *g* for 15 min at 4 ℃. Thereafter, supernatants were divided into aliquots, and were stored at −80 °C for further analysis of parameters related to brain molecular functions. The remaining part was frozen in liquid nitrogen and immediately stored at −80 °C. This aliquot was used for precipitation with acetonitrile to proceed the neuropeptide assessment with Milliplex MAP assay.

### 2.4. Hematological Test

The hematological parameters were measured using an automated hematology analyzer Sysmex K-1000D (Sysmex, Tokyo, Japan).

### 2.5. Iron Assessments 

Serum iron, total iron-binding capacity (TIBC), and transferrin saturation were determined using Sigma Diagnostics Iron and TIBC reagents (Sigma, St Louis, MO, USA). Concentrations of serum ferritin (µg·L^−1^) and serum hepcidin (Hepcidin-25 ng·mL^−1^) were determined using a rat ELISA Kit (Biovendor Gmbh, Heidelberg, Germany) for TIBC and another commercial kit for serum ferritin (DRG Instruments GmbH, Marburg, Germany). 

### 2.6. Dopamine

Dopamine levels in brain homogenate were measured using a commercial enzyme immunoassay kit (MyBioSource, San Diego, CA, USA). Once the tissues were homogenized, the resulting suspension was subjected to ultrasonication. After that, the homogenates were centrifugated for 15 min at 1500× *g*. Measurements in duplicate were used to determine intra-assay variability.

### 2.7. Serotonin

The level of serotonin in brain homogenate was determined using a commercially available enzyme immunoassay ELISA Kit (MyBioSource, San Diego, CA, USA). One hundred microliters of the homogenate were added to duplicate wells in the ELISA plate, which was then processed according to the directions of the manufacturer.

### 2.8. MAO-A and MAO-B

To determine monoamine oxidase A and B (MAO-A and MAO-B) brain homogenate levels, a commercial rat ELISA kit was performed (Wuhan Fine Biological Technology Co., Ltd., Wuhan, China). After brain homogenization, the resulting suspension was sonicated with an ultrasonic cell disrupter to break the cell membranes. After that, the homogenates were centrifugated for 5 min at 5000× *g*. Then, the concentration of MAO-A and MAO-B was determined by measuring the absorbance at 450 nm (Bio-tek, Winooski, VT, USA).

### 2.9. Irisin

Irisin levels in brain homogenate were measured using a commercial kit (Wuhan Fine Biological Technology Co., Ltd., Wuhan, China). To further break the cells, the homogenate suspension was sonicated with an ultrasonic cell disrupter. The homogenates were then centrifuged for 5 min at 5000× *g*, and absorbance from each sample was measured in duplicate using a spectrophotometric microplate reader at a wavelength of 450 nm (Bio-tek, Winooski, VT, USA).

### 2.10. Synaptophysin

Synaptophysin in brain homogenate was measured using a commercial ELISA kit (USCN Life Science, Euromedex, Souffelweyersheim, France). After addition of the substrate solution, the intensity of color developed is reverse proportional to the concentration of synaptophysin in the sample. Plates were read spectrophotometrically (Bio-tek, Winooski, VT, USA) at 450 nm.

### 2.11. Neuropeptides Assessment

α-Melanocyte-stimulating hormone (α-MSH), β-endorphin, neurotensin, oxytocin, and substance P on acetonitrile-precipitated brain homogenate extracts were determined, using the RMNPMAG-83K Milliplex MAP Rat Neuropeptide Magnetic Bead Panel (Millipore Corporation, City, TX, USA). The plate was read on a LABScan 100 analyzer (Luminex Corporation, Austin, TX, USA) with xPONENT software (Luminex Corporation, Austin, TX, USA) for data acquisition. Neuropeptides on acetonitrile-precipitated brain homogenate extracts were determined by comparing the mean of duplicate samples with the standard curve for each assay.

### 2.12. Statistical Analysis

These analyses were carried out using the SPSS computer program (version 24.0, 2016, SPSS Inc., Chicago, IL, USA). To test differences between groups (normal Fe versus low Fe) during the PEP, Student’s *t* test was used. Individual means were tested by pairwise comparison with Tukey’s multiple comparison test when main effects and their interactions were significant. Two-way ANOVA was performed to determine the effects of type of diet, anemia, and iron content in the diet. *p* < 0.05 was set as significant.

## 3. Results

Iron deprivation during the pre-experimental period markedly decreased all the hematological parameters in the anemic group compared to the controls (*p* < 0.001); meanwhile, red cell distribution width, platelets count, total iron-binding capacity, and hepcidin were higher (*p* < 0.001), and the white blood cell count remained unchanged (Table 1). 

With both fermented milk-based diets, the hematological parameters showed a recovery after supplying the normal-Fe or Fe-overload fermented milk-based diets (EP). Serum hepcidin decreased in control and anemic animals fed fermented goat milk (normal iron content or Fe overload) in comparison with fermented cow milk (*p* < 0.001). Serum iron increased in the Fe-overload groups in all experimental conditions (*p* < 0.01). Fe overload also increased hemoglobin (*p* < 0.001), hematocrit (*p* < 0.01), total iron-binding capacity (*p* < 0.01), transferrin saturation (*p* < 0.01), and serum ferritin (*p* < 0.01) (Table 2).

IDA decreased most of the parameters related to brain molecular functions, namely dopamine (*p* < 0.05), MAO-A, oxytocin, irisin, α-MSH, and β-endorphin (*p* < 0.001), while it increased synaptophysin (*p* < 0.001) (Table 3).

With regard to the brain molecular function parameters studied after IDA recovery (EP), Table 4 shows that 30 days after supplying the fermented milk-based diets, fermented goat milk consumption increased dopamine in both groups of animals with normal Fe with respect to fermented cow milk (*p* < 0.01), while it decreased this parameter in the Fe-overload control animals (*p* < 0.01). Fermented goat milk decreased MAO-A in both groups of animals: those in the Fe-overload group (*p* < 0.001) and in the control group fed normal Fe (*p* < 0.05). It also decreased the MAO-B in both groups of animals fed fermented goat milk with normal Fe (*p* < 0.001) levels. Synaptophysin increased in all the groups fed fermented goat milk either, with normal Fe or Fe overload (*p* < 0.001 for the control groups, and (*p* < 0.01 for the anemic groups, except for anemic animals fed fermented goat milk with normal Fe, in which we observed a reduction (*p* < 0.01). α-MSH increased in all the groups fed fermented goat milk (*p* < 0.001 for normal Fe and *p* < 0.01 for Fe overload). 

Anemia decreased the dopamine in animals fed fermented goat or cow milk with Fe overload (*p* < 0.05 and *p* < 0.001 respectively), MAO-A and MAO-B in the animals fed fermented cow milk (*p* < 0.001 for normal Fe and *p* < 0.05 for Fe overload), and goat milk with Fe overload (*p* < 0.001 MAO-A and *p* < 0.05 MAO-B). Anemia increased neurotensin in the normal-Fe groups fed fermented cow milk (*p* < 0.001). Oxytocin increased in the anemic animals fed fermented cow milk with normal Fe (*p* < 0.01), and decreased in animals fed both types of fermented milk with Fe overload (*p* < 0.001). Serotonin increased in anemic animals fed both milk-based diets with normal Fe (*p* < 0.001) in comparison with control animals. Synaptophysin increased in the anemic animals fed fermented cow milk (*p* < 0.01), and decreased in the anemic animals fed fermented goat milk (*p* < 0.001) in both normal and Fe overload. Anemia decreased α-MSH levels in all the groups fed both milk-based diets either with normal Fe or Fe overload (*p* < 0.001). Anemia decreased β-endorphin levels in the animals fed both fermented milks with normal Fe (*p* < 0.001) (Table 4).

Fe overload increased dopamine levels in both groups of animals fed fermented cow milk diet (*p* < 0.01), and increased MAO-A levels in the control groups fed both milk-based diets (*p* < 0.001), while it decreased this parameter in the anemic group fed fermented goat milk (*p* < 0.001). Fe overload also increased MAO-B levels in all the groups fed both types of fermented milk (*p* < 0.01), except for the anemic groups fed fermented cow milk. Fe overload increased neurotensin in the control group fed fermented cow milk (*p* < 0.01), and decreased this parameter in the anemic group fed the same diet (*p* < 0.01). For the mice in the Fe-overload groups, oxytocin increased in control animals fed both fermented milk diets (*p* < 0.01) and decreased in anemic animals fed both fermented milk diets (*p* < 0.001). Fe overload caused a marked reduction in serotonine levels in control and anemic rats fed both milk-based diets (*p* < 0.001). Synaptophysin decreased in the control groups and anemic rats fed fermented cow milk (*p* < 0.001), and increased in the animals fed fermented goat milk (*p* < 0.001). Fe overload increased α-MSH levels in the groups fed fermented cow milk (*p* < 0.001), and decreased this hormone in the groups fed fermented goat milk (*p* < 0.01). β-Endorphin decreased in the control animals fed fermented cow or goat milk with Fe overload (*p* < 0.001) (Table 4).

## 4. Discussion

A better characterization of the events in the pathophysiology of IDA and the influence on nervous system molecular functions and homeostasis during the recovery of this deficiency would led to new nutritional strategies improving brain molecular functions and the other deleterious effects of the pathology. The animal model used in the current study simulates physiological deficiencies to iron and anemia recovery, and therefore argues that the response to brain functions can be modulated by dietary components.

The results of the current study reveal a clear impairment in some brain molecular functions due to IDA, because most of the parameters studied were impaired. ID manifests as alterations in cognitive function, behavior, and mood [19]. Iron is a cofactor of many metabolic processes as well as the synthesis of aminergic neurotransmitters; it plays a major function in brain development, and has a key role in myelinogenesis and synaptogenesis. Several mechanisms regarding the influence of iron deficiency on brain functions have been reported: the decrease in oxygen-carrying capacity of the blood could result in hypoperfusion of the brain, which could lead to an imbalance in oxidative/antioxidant status and inflammatory responses, causing neurodegenerative processes [20]. Furthermore, anemia induces renal changes, leading to lower erythropoietin levels as well as increasing the risk of neural degeneration, as this hormone has neuroprotective effects in situations of hypoxia [21]. Thus, IDA induces pathological changes in brain tissue and vessels, leading to reduced oxygen transportation as well as impaired synaptic functioning [22]. 

Dopamine levels have been heavily examined in patients suffering from IDA, and it is well established that this neurotransmitter is implicated in learning, memory, and attention, as well as several hormonal pathways, stress responses, addiction, and emotional behavior [23]. IDA leads to diminished central dopaminergic transmission and receptor trafficking, with the D2 receptor particularly affected [24]. Other evidence of biochemical abnormalities during IDA include decreased concentrations of thyroid hormones, as we have previously reported [25], increasing the levels of circulating catecholamines. This involves beta adrenergic receptors and affects the availability of glucose, impairing synaptic plasticity and changes in dendritic structure that lead to a loss of neurons [26], indicating that IDA can alter neurotransmitter metabolism.

Surprisingly, milk-based diets have really different effects on dopamine and serotonin, which are both monoamines that have iron-dependent synthesis pathways. In general, there is consensus in the scientific literature reporting a negative effect of ID on dopamine functions and synthesis [27], which coincides with our results during the anemia induction. However, conflicting results exist on the effects of ID on serotonin levels in rats. ID decreases serotonin due to a down-regulation of several biosynthesis pathways in young rats [28]. On the contrary, an increase in serotonin levels during ID has been reported in adults, reflecting a down-regulation of serotonin metabolism [29]. Serotonin transporters are reduced in the striatum of ID mice [30]. Additionally, gestational ID reduces serotonin uptake by synaptic vesicles in offspring, which is a process that can be normalized after four weeks of iron replenishment [31]. However, in other studies, ID had no effect on serotonin levels or metabolism in newborns or adults [32], and serotonin levels in the prefrontal cortex of the ID rats did not differ from controls [33]. These reported results in serotonin homeostasis appear conflicting, and hint at underlying additional mechanisms of the iron–monoamine relationship [2]. Therefore, although ID did not changed serotonin levels, the differences during iron repletion could be attributed to the different behavior of serotonin mentioned above, and the enhancement of the digestive and metabolic utilization of iron in fermented goat milk [14]. Moreover, a limitation of the study is the lack of a regional analysis of the brain molecules studied, which could help to specify the effect of fermented dairy products on brain functions, and could explain these results.

It has been reported that irisin promotes the differentiation of human embryonic stem cell-derived neural cells into neurons, as well as increased mature neuronal and astrocyte markers together with the improved expression of neurotrophic factors in the brain [34]. Therefore, the decrease of irisin levels in this study due to IDA reveals impairment in the molecular mechanisms driving neuron homeostasis. Moreover, the decrease recorded in MAO-A can be explained because iron is a key factor for MAO activity, and monoamine neurotransmitter synthesis requires the iron-dependent enzymes tyrosine and tryptophan hydroxylase, which is a finding that has been correlated with later behavioral consequences in juvenile monkeys, influencing brain function [35]. IDA also decreased oxytocin, which is a hypothalamic neuropeptide involved in regulating social behavior, and has a key role in physiological conditions and brain diseases. It has been reported that CO exerts an inhibitory tone on oxytocin secretion [36], and it is well known that CO output is increased during IDA. Additionally, IDA also decreased α-MSH which has been proven as an anti-inflammatory and neuroprotective hormone in animal studies [37] and β-endorphin, which has an important role in the development of the non-synaptic or paracrine communication between neurons [38], revealing the impairment of brain molecular functions.

On the other hand, synaptophysin increased during IDA. This interesting result can be explained because synaptobrevin II is a vesicular protein receptor that is essential for neurotransmitter release; therefore, its correct trafficking to synaptic vesicles is critical to render them fusion-competent. Synaptophysin binds to synaptobrevin II in the synaptic vesicles and facilitates its retrieval during endocytosis. Under physiological conditions, the expression of synaptophysin in a 1:2 ratio with synaptobrevin II is sufficient to fully rescue normal synaptobrevin II trafficking. The balance between synaptophysin and synaptobrevin II is critical for the exocytotic release of neurotransmitters [39]. Since as previously mentioned, anemia is associated with an impairment in cerebral blood flow and oxygen metabolism, as well as low neurotransmitter release [7], the overexpression of synaptophysin could be a compensatory mechanism to cope with the low neurotransmitter release from the synaptic vesicles during this condition.

In general, an improvement of nervous system molecular functions has been observed after anemia recovery with fermented goat milk (including dopamine, oxytocin, serotonin, α-MSH, and synaptophysin), which can be explained by several factors, including the better recovery of IDA with this dairy product.

Previous studies of our research group showed an increased expression of some key iron metabolism proteins such as duodenal cytochrome b, divalent metal transporter 1, and ferroportin 1 in rats fed fermented goat milk compared to fermented cow milk. These proteins enable overcoming the effects of IDA, increasing iron bioavailability in target organs and efficient iron repletion after IDA [14], and have significant implications in the brain molecular mechanisms related to cognitive functions. 

On the other hand, synaptophysin is considered a reliable biomarker for synaptic density and synaptogenesis [40]. Synaptophysin is also correlated with a loss or increase in synaptic densities in studies of aging and neurodegenerative disorders [41]. In the current study, the production of synaptophysin was significantly increased in the rats consuming fermented goat milk, because as previously reported [42], the production of neurotrophic factors, as well as the survival of neuronal synapses and the retention of cognitive function is increased when the inflammation is suppressed. 

In this sense, we have previously reported that in control and anemic rats, interleukin (IL)-1β, IL-2, IL-12p70, IP-10 and tumour necrosis factor (TNF)-α (pro-inflammatory cytokines) decrease after fermented goat milk consumption, and levels of IL-4, IL-13 and IL-10 (pro-inflammatory cytokines) [43] increase. These results are due to the better nutritional characteristics of fermented goat milk, in comparison with fermented cow milk, playing a potential role of this dairy product as a high nutritional value food with anti-inflammatory properties. It has also been reported that TNF-α produced by microglia exacerbates the neurodegenerative diseases [44]. In the brain, microglia mainly regulate immunological phenomena; as a result, it seems that fermented goat milk may regulate also the microglial inflammatory response, leading to a suppression of the inflammatory signaling and neuronal degeneration. Chronic inflammation in the brain exacerbates the pathological condition and cognitive functions decline in many neurodegenerative processes, which is due to neurotrophic factors that are suppressed by inflammation and are toxic to neurons [45,46].

Increased awareness of the role of oxidative stress in the pathogenesis of neurodegenerative processes has highlighted the issue of whether oxidative damage is a fundamental step in the pathogenesis or instead results from disease-associated pathology. Recently, it has been reported that oxidative damage results in amyloid deposition in the brain, resulting in neuronal cell death and neurodegenerative diseases [47,48]. In this sense, we have previously reported [49,50] that fermented goat milk increased some antioxidant enzymes in brain tissue as well as total antioxidant status and melatonin, even in the case of Fe overload. These increases limit the oxidative damage to the brain biomolecules (lipids, protein DNA, prostaglandins) and protect the nervous tissue of the oxidative-induced cell death that induces neurodegenerative processes.

In the current study, iron overload increased dopamine levels in the brain with a fermented cow milk based-diet, and it has been previously reported that iron accumulation relates to dopamine, comprising a toxic couple that is reliant on interacting with other biomolecules, and causes selective neurodegeneration in some areas of the brain [51]. MAO-A levels were increased in control animals consuming both types of fermented milk with iron overload; an enzyme catalyzes the oxidative deamination of monoamine neurotransmitters, increasing the production of reactive oxygen species and oxidative stress, which is potentially a risk factor for neuronal loss and neurodegenerative disorders [52]. Iron excess also reduced β-endorphin in the control animals, which has several activities such as analgesic, immunostimulatory, stress busting, and anti-inflammatory, as well as having a key role in the adequate neurological responses [53]. These deleterious effects of iron overload are in accordance with previous reports revealing that iron supplementation results in brain accumulation and subsequent toxicity, increasing oxidative damage up to a level that natural defenses fail, and at which neuronal apoptotic rates are exacerbated [54].

We have previously reported that except for alanine, in which the differences were not statistically significant, all other amino acids were higher in fermented goat milk than in fermented cow milk, including glycine threonine and tyrosine [15], and it has been reported that orally administered β-lactopeptide of glycine-threonine-tryptophan-tyrosine inhibits the activity of monoamine oxidase in the brain [55]. It has also been reported that the MAO inhibitor reduces reactive oxygen species and suppresses some neurodegenerative diseases [56]. In addition, the inhibitory activity of MAO reduces the activation of nuclear factor kappa B (NF-κB) and suppresses NF-κB-regulated pro-inflammatory responses [57], which supports the results obtained with the animals fed fermented goat milk. In the current study, an increase in α-MSH was also recorded in all groups fed fermented goat milk. α-MSH is a hormone that functions as a neurotransmitter and neuromodulator; it is involved in significant neuronal circuitry, and is also a mediator of immunity and inflammation. At the molecular level, these effects of α-MSH are mediated via the inhibition of the activation of transcription factors such as NF-κB [58], reducing once more the pro-inflammatory responses in the nervous system and suppressing some pathways that lead to neurodegenerative environments.

## 5. Conclusions 

In conclusion, by using an animal model of severe iron deficiency, the current study has showed a relation between iron status and brain molecular parameters related to key functions during anemia instauration and recovery. The alterations recorded in the biomarker-related brain functions studied may result in impairments in behavioral and cognitive functions during severe iron deficiency anemia. In addition, the results of the current study reveal that in general, during anemia recovery, a fermented goat milk-based diet, normal iron, or iron overload inhibits MAO activities, and increases serotonin, synaptophysin, and α-MSH levels. It also has been implicated in the suppression of inflammatory responses, an improvement in iron metabolism, and a reduction of evoked oxidative stress, as observed in previous studies. Taken together, all these results suggest a potential neuroprotective effect of fermented goat milk, which could enhance brain molecular functions, although further studies are needed to confirm these findings.

## Figures and Tables

**Table 1:** Hematological parameters from control and anemic rats (pre-experimental period).

	Normal-Fe Control Group (*n* = 50)	Low-Fe Anemic Group (*n* = 50)
Hemoglobin concentration (g·L^−1^)	130.62 ± 2.77	60.46 ± 2.77 *
Red blood cells (10^12^·L^−1^)	7.11 ± 0.22	3.19 ± 0.30 *
Hematocrit (%)	41.13 ± 1.03	13.12 ± 1.33 *
Mean corpuscular volume (fL)	54.92 ± 0.52	37.21 ± 0.37 *
Mean corpuscular hemoglobin (pg)	20.01 ± 0.15	14.22 ± 0.53 *
Mean corpuscular hemoglobin concentration (g·dL^−1^)	36.12 ± 0.37	30.56 ± 0.73 *
Red cell distribution width (%)	16.33 ± 0.45	19.67 ± 0.35 *
Platelets (10^9^·L^−1^)	731 ± 70.11	2123 ± 112 *
White blood cells (10^9^·L^−1^)	8.32 ± 0.41	8.45 ± 0.59
Lymphocytes (10^6^·mL^−1^)	7.76 ± 0.51	5.62 ± 0.77 *
Fe (µg·L^−1^)	1342 ± 97.31	607 ± 55.22 *
Total iron-binding capacity (µg·L^−1^)	2623 ± 179	18002 ± 539 *
Transferrin saturation (%)	49.22 ± 5.86	4.01 ± 0.45 *
Ferritin (µg·L^−1^)	79.54 ± 2.23	49.12 ± 1.58 *
Hepcidin (ng·mL^−1^)	13.25 ± 0.32	15.42 ± 0.68 *

Data are shown as the mean values ± Standard error of the mean. * Significantly different from the control group (*p* < 0.001, Student’s *t* test).

**Table 2:** Hematological parameters from control and anemic rats fed for 30 days with fermented cow or goat milk-based diets with normal Fe content or Fe overload (*n* = 10 animals per group).

	Fe Content	Fermented Cow Milk Diet		Fermented Goat Milk Diet		Two-Way ANOVA		
		Control Group	Anemic Group	Control Group	Anemic Group	Diet	Anemia	Fe Content
Hemoglobin concentration (g·L^−1^)	Normal	127.73 ± 2.35	128.42 ± 2.43	131.91 ± 2.44	130.01 ± 2.39	NS ^1^	NS	<0.001
	Overload	141.97 ± 2.53 ^C^	140.18 ± 2.87 ^C^	141.43 ± 2.77 ^C^	145.62 ± 2.92 ^BC^	<0.05	NS	
Red blood cells (10^12^·L^−1^)	Normal	7.21 ± 0.18	7.16 ± 0.20	7.35 ± 0.22	7.32 ± 0.19	NS	NS	<0.05
	Overload	7.01 ± 0.19	7.19 ± 0.19	8.12 ± 0.28 ^AC^	7.21 ± 0.22	<0.01	NS	
Hematocrit (%)	Normal	40.22 ± 1.12	39.22 ± 1.01	41.87 ± 1.41 ^A^	43.05 ± 1.11 ^B^	<0.01	NS	<0.01
	Overload	39.87 ± 1.25	45.11 ± 2.34 ^C^	44.75 ± 1.51 ^AC^	44.91 ± 1.41 ^C^	<0.05	NS	
Mean corpuscular volume (fL)	Normal	57.28 ± 0.54	55.28 ± 0.53	57.41 ± 0.57	55.22 ± 0.54	NS	NS	NS
	Overload	56.90 ± 0.60	53.39 ± 0.54	56.62 ± 0.54	56.33 ± 0.51 ^B^	<0.05	NS	
Platelets (10^9^·L^−1^)	Normal	935.62 ± 72.11	961.53 ± 67.33	929.21 ± 78.11	937.32 ± 68.53	NS	NS	NS
	Overload	939.22 ± 71.24	963.29 ± 70.21	936.12 ± 79.76	942.12 ± 70.25	NS	NS	
Serum Fe (µg·L^−1^)	Normal	1323 ± 81.88	1347 ± 85.33	1362 ± 88.22	1332 ± 91.13	NS	NS	<0.01
	Overload	1576 ± 98.56 ^C^	1592 ± 96.25 ^C^	1552 ± 97.89 ^C^	1569 ± 95.76 ^C^	NS	NS	
Total iron-binding capacity (µg·L^−1^)	Normal	2782 ± 153	2788 ± 142	2791 ± 143	2786 ± 152	NS	NS	<0.01
	Overload	3151 ± 167 ^C^	3234 ± 171 ^C^	3241 ± 166 ^C^	3188 ± 169 ^C^	NS	NS	
Transferrin saturation (%)	Normal	46.22 ± 0.91	45.50 ± 0.87	46.79 ± 0.78	46.89 ± 0.91	NS	NS	<0.01
	Overload	47.85 ± 1.21 ^C^	47.62 ± 1.12 ^C^	48.96 ± 1.11 ^C^	48.79 ± 1.07 ^C^	NS	NS	
Serum ferritin (µg·L^−1^)	Normal	83.11 ± 1.56	83.77 ± 1.29	84.31 ± 1.65	82.24 ± 1.82	NS	NS	<0.01
	Overload	86.95 ± 1.88 ^C^	85.98 ± 1.76 ^C^	86.96 ± 1.83 ^C^	87.03 ± 1.79 ^C^	NS	NS	
Serum hepcidin (ng·mL^−1^)	Normal	16.21 ± 0.61	16.37 ± 0.53	14.11 ± 0.58 ^A^	14.33 ± 0.61 ^B^	<0.01	NS	NS
	Overload	16.42 ± 0.58	16.39 ± 0.51	15.07 ± 0.58^A^	14.22± 0.57^B^	<0.01	NS	

Data are shown as the mean values ± SEM. ^1^ NS, not significant. ^A^ Mean values from control group fed with fermented cow milk diet were significantly different (*p* < 0.05, Tukey’s test). ^B^ Mean values from the anemic group fed with fermented cow milk diet were significantly different (*p* < 0.05, Tukey’s test). ^C^ Mean values from the corresponding group fed with normal Fe content were significantly different (*p* < 0.05, Tukey’s test).

**Table 3:** Brain molecular function parameters in brain homogenate (pg mL^−1^) from control and anemic rats (pre-experimental period).

	Normal-Fe Control Group (*n* = 10)	Low-Fe Anemic Group (*n* = 10)
Dopamine	1970.00 ± 180.21	1560.01 ± 90.05 *
Serotonin	9179.0 ± 174.9	9409.0 ± 1813.5
MAO-A	7856.3 ± 225.2	6622.3 ± 212.7 **
MAO-B	267.96 ± 25.85	219.85 ± 10.81
Neurotensin	513.40 ± 48.18	442.61 ± 37.63
Oxytocin	249.71 ± 21.22	181.85 ± 17.86 **
Irisin	21.17 ± 1.19	16.74 ± 0.90 **
Synaptophysin	771.79 ± 36.97	1121.37 ± 28.62 **
α-MSH	616.34 ± 20.52	200.58 ± 44.29 **
β-Endorphin	2431.5 ± 126.0	1308.3 ± 186.6 **

Data are shown as the mean values ± SEM. Significantly different from the control group (* *p* < 0.05, ** *p* < 0.001, Student’s *t* test). MAO, Monoamine oxidase; α-MSH, α-Melanocyte-stimulating hormone.

**Table 4:** Brain molecular function parameters in brain homogenate (pg mL^−1^) from control and anemic rats fed for 30 days with fermented cow or goat milk-based diets with normal Fe content or Fe overload (*n* = 10 animals per group).

	Fe Content	Fermented Cow Milk Diet		Fermented Goat Milk Diet		Two-Way ANOVA		
		Control Group	Anemic Group	Control Group	Anemic Group	Diet	Anemia	Fe Content
Dopamine	Normal	1210.00 ± 40.01	980.14 ± 30.07	1710.10 ± 16.07 ^A^	1320.11 ± 11.23 ^B^	<0.01	NS	<0.05
	Overload	3130.02 ± 22.14 ^D^	1440.21 ± 30.12 ^CD^	1400.02 ± 60.04 ^A^	1260 ± 40.33 ^C^	<0.01	< 0.01	
Serotonin	Normal	33651.9 ± 3334.5	61341.9 ± 5820.1 ^C^	39278.9 ± 4571.7	82050.5 ± 2995.1 ^BC^	<0.05	<0.001	<0.001
	Overload	26346.4 ± 2943.3 ^D^	29564.3 ± 2806.3 ^D^	20981.4 ± 852.7 ^D^	23688.0 ± 962.7 ^D^	NS	NS	
MAO-A	Normal	8220.1 ± 261.5	7279.3 ± 298.6 ^C^	7390.3 ± 211.9 ^A^	6716.7 ± 483.3 ^B^	<0.05	<0.05	<0.01
	Overload	13935.1 ± 282.3 ^D^	7152.6 ± 498.0 ^C^	8403.7 ± 353.4 ^AD^	4001.8 ± 168.3 ^BCD^	<0.001	<0.001	
MAO-B	Normal	260.96 ± 10.81	237.89 ± 11.57 ^C^	215.13 ± 10.10 ^A^	208.93 ± 10.07 ^B^	<0.001	<0.05	<0.01
	Overload	327.70 ± 9.56 ^D^	259.96 ± 6.68 ^C^	305.24 ± 9.01 ^D^	267.52 ± 7.90 ^CD^	NS	<0.01	
Neurotensin	Normal	317.98 ± 11.94	405.37 ± 18.23 ^C^	336.36 ± 21.37	373.21 ± 10.15	NS	<0.05	<0.05
	Overload	360.77 ± 12.67 ^D^	345.61 ± 12.80 ^D^	355.79 ± 10.39	341.56 ± 9.98	NS	NS	
Oxytocin	Normal	145.57 ± 9.27	173.42 ± 33.90 ^C^	182.41 ± 7.27 ^A^	177.74 ± 1.87	<0.05	<0.05	<0.001
	Overload	217.27 ± 3.58 ^D^	86.95 ± 0.13 ^CD^	225.52 ± 3.94 ^D^	89.85 ± 1.57 ^CD^	NS	<0.01	
Irisin	Normal	16.75 ± 0.70	15.96 ± 0.35	19.18 ± 1.26	17.24 ± 1.03	NS	NS	NS
	Overload	19.32 ± 0.60	16.66 ± 0.85	17.58 ± 0.79	15.64 ± 0.70	NS	NS	
Synaptophysin	Normal	836.97 ± 31.40	907.33 ± 27.57 ^C^	1091.02 ± 26.92 ^A^	730.09 ± 20.44 ^BC^	<0.001	<0.01	<0.001
	Overload	552.01 ± 40.56 ^D^	789.02 ± 49.59 ^CD^	1290.56 ± 51.89 ^AD^	883.40 ± 24.74 ^BCD^	<0.001	<0.001	
α-MSH	Normal	310.87 ± 14.95	86.28 ± 1.38 ^C^	1162.05 ± 9.07 ^A^	494.28 ± 22.64 ^BC^	<0.001	<0.001	<0.001
	Overload	669.74 ± 33.48 ^D^	251.69 ± 18.04 ^CD^	726.85 ± 21.86 ^A D^	312.54 ± 9.40 ^BC D^	<0.01	<0.001	
β-Endorphin	Normal	2426.5 ± 79.4	744.3 ± 39.9 ^C^	2551.9 ± 131.7	671.3 ± 36.4 ^C^	NS	<0.01	<0.05
	Overload	741.6 ± 41.3 ^D^	688.6 ± 40.9	1344.2 ± 71.5 ^AD^	640.1 ± 34.0 ^C^	<0.05	NS	

Data are shown as the mean values ± SEM. NS, not significant. ^A^ Mean values from control group fed a fermented cow milk diet were significantly different (*p* < 0.05, Tukey’s test). ^B^ Mean values from anemic group fed a fermented cow milk diet were significantly different (*p < 0.05*, Tukey’s test). ^C^ Mean values from the corresponding group of anemic animals were significantly different (*p* < 0.05, Tukey’s test). ^D^ Mean values from the corresponding group fed with normal Fe content were significantly different (*p* < 0.05, Student’s *t* test). MAO, Monoamine oxidase; α-MSH, α-Melanocyte-stimulating hormone.
